# Drug Incompatibilities and Complex Assemblies: Let Us Remain Vigilant!

**DOI:** 10.3390/ph18050626

**Published:** 2025-04-25

**Authors:** Cordélia Salomez-Ihl, Anthony Martin Mena, Marie-Carmen Molina, Romane Chapuis, Marjorie Durand, Sébastien Chanoine, Julien Leenhardt, Philippe Py, Marie-Dominique Brunet, Yung-Sing Wong, Marie Chevallier, Bertrand Décaudin, Pascal Odou, Pierrick Bedouch, Roseline Mazet

**Affiliations:** 1 Pharmacy Department, Grenoble University Hospital, F-38000 Grenoble, France; rchapuis@chu-grenoble.fr (R.C.); mdurand1@chu-grenoble.fr (M.D.); schanoine@chu-grenoble.fr (S.C.); jleenhardt@chu-grenoble.fr (J.L.); ppy@chu-grenoble.fr (P.P.); mdbrunet@chu-grenoble.fr (M.-D.B.); pbedouch@chu-grenoble.fr (P.B.); rmazet@chu-grenoble.fr (R.M.); 2 THEMAS, TIMC-IMAG UMR CNRS 5525, Faculty of Pharmacy and Medicine, University Grenoble Alpes, F-38000 Grenoble, France; mchevallier3@chu-grenoble.fr; 3 ULR 7365-GRITA-Groupe de Recherche sur les Formes Injectables et les Technologies Associées, Faculty of Pharmacy and Medicine, University Lille, F-59000 Lille, France; anthony.martin-mena@univ-lille.fr (A.M.M.); bertrand.decaudin@univ-lille.fr (B.D.); pascal.odou@univ-lille.fr (P.O.); 4 DPM, UMR CNRS 5063, ICMG FR 2607, University Grenoble Alpes, F-38000 Grenoble, France; marie-carmen.molina@univ-grenoble-alpes.fr (M.-C.M.); yung-sing.wong@univ-grenoble-alpes.fr (Y.-S.W.); 5 INSERM, LRB, UMR 1039, University Grenoble Alpes, F-38000 Grenoble, France; 6 Neonatology Department, Grenoble University Hospital, F-38000 Grenoble, France; 7 Pharmacy Department, Lille University Hospital, F-59000 Lille, France

**Keywords:** drug incompatibilities, drug infusion systems, neonatal care units, parenteral nutrition, ganciclovir, multi-lumen infusion devices

## Abstract

**Background/Objectives**: Multi-lumen devices that limit physicochemical incompatibilities (PCIs) are frequently used in neonatal intensive care units where premature infants receive numerous infusions. The aim of the study was to investigate a PCI that occurred despite the use of a device of this type (EDELVAISS^®^ Multiline NEO, Doran International, Toussieu, France). **Case Summary**: A 7-week-old preterm infant received ganciclovir at therapeutic dosage for cytomegalovirus (CMV) infection. After the fifth administration of ganciclovir, a PCI occurred, leading to a white precipitate. The peripheral inserted central catheter (PICC) (PREMICATH^®^2Fr, Vygon, Ecouen, France) had to be replaced. Laboratory reproduction of the administrations during 72 h, nuclear magnetic resonance (NMR) analysis and particle counting were carried out to analyse the occurrence of events leading to PCIs. The precipitate was linked to a PCI of parenteral nutrition associated with a dilution error of ganciclovir (omission of a 10-fold dilution step, resulting in ganciclovir being administered at 30 mg/L instead of 3 mg/L). Due to the presence of lipids in the parenteral nutrition, visual detection of the white precipitate was difficult. **Conclusions**: Multi-lumen infusion devices limit but do not prevent the occurrence of PCIs, particularly in the event of a preparation error. Despite the use of this type of device, great vigilance is still required, particularly with regard to prescription analysis and reconstitution procedures.

## 1. Introduction

Physicochemical incompatibilities (PCIs) between intravenous drugs are one of the problems frequently encountered in everyday practice, particularly in critical care units, with potentially major clinical consequences [1,2,3,4]. Patients in paediatric intensive care units are also concerned, especially as they receive numerous parenteral medications [5]. These drug incompatibilities are physicochemical reactions that can lead to a loss of the active ingredient(s) but may also result in the formation of a precipitate visible to the naked eye, which is generally associated with a non-visible particulate load [6]. The consequences of these PCIs include, on the one hand, the obstruction of the infusion line and, on the other hand, the cause of various clinical consequences such as thromboses, phlebitis, or respiratory distress syndromes [7,8,9].

These PCIs have already been described as potentially linked to administration or preparation errors, with the need to optimise working conditions to reduce the proportion of errors, leading to these PCIs [10]. While all therapies may potentially be involved, parenteral nutrition (PN) is a form of intravenous therapy, which in its own right can be responsible for PCIs [11,12,13].

To limit the occurrence of these PCIs, various strategies have been implemented in healthcare services [14]. First, the use of double-entry cross-tables allows for the determination of compatibility between two medications [15]. Additionally, the use of separate infusion lines or sequential infusion are two interesting strategies, but this is not always feasible in intensive care units [16,17,18]. Finally, various modifications can be made to the infusion line, such as adding inline filters or using medical devices with low residual volume. Multi-lumen catheters are recommended in critical care units [19,20,21]. An EDELVAISS^®^ Multiline Neo device is a multi-access infusion device specially designed for the neonatal population. This device has five ports connected to separate lumens within a single 90 cm tube. A sixth port, called an “access port”, with a small residual volume of 0.40 mL, is intended for emergency situations and direct intravenous injections, allowing for rapid intervention when needed. Valid for continuous use for up to 21 days, the EDELVAISS^®^ Multiline Neo device is designed to be placed outside the incubator to minimise handling inside it. The common volume is considerably reduced, as the tubing only joins at the terminal end of the device. The reduced contact volume limits the contact time and, therefore, reduces the risk of PCIs [11].

The use of multi-light devices does not, however, exempt the user from a medical and pharmaceutical analysis of the prescription in order to identify possible PCI, as recommended in all clinical services. Indeed, a common volume persists at the end of the device. The aim of this work is to study the case of a PCI that occurred in neonatal intensive care despite the use of an EDELVAISS^®^ Multiline Neo device (Doran International, Toussieu, France) in order to draw lessons for clinical practice.

## 2. Case Presentation

We report the case of a premature child, born before 26 weeks of gestational age. At day 40 post-natal, a cytomegalovirus (CMV) infection was diagnosed with thrombopenia, hyperechogenicity of the thalamostriate vessels, and colitis. This CMV infection was treated with ganciclovir at a therapeutic dosage (Table 1). Detecting and treating this pathology is a major challenge in the care of newborn babies, particularly because of the potentially associated adverse effects, like neurologic injuries and hearing loss [22].

At the same time, the patient received parenteral nutrition (PN) of a suitable composition, which was re-evaluated every day according to the evolution of its nutritional needs (Table 1). A multi-lumen infusion device (EDELVAISS^®^ Multiline Neo device, Doran International, Toussieu, France) was used to limit PCIs. Despite the use of this device, the obstruction of its PICC was reported. It had to be replaced, which required a new technical procedure and anaesthesia, adding risks, particularly infectious. The medical team investigated the sequence of events that led to this obstruction of the PICC and its emergency replacement.

First, a survey of the nursing team and a retrospective study of prescriptions were conducted to detail the set-up and drug administration conditions to the neonate (concentrations, administration scheme, sequence, and flow rate presented in Table 1) following the installation of the PICC. As the times of administration were recorded, it was possible to construct a precise description of the therapies received by the patient.

According to the summary of product characteristics, ganciclovir (CYMEVAN^®^ 500 mg, Cheplapharm Arzneimittel GmbH, Greifswald, Germany) is reconstituted with 10 mL of water for injection (Laboratoire Aguettant, Lyon, France) into the Neonatal Intensive Care Unit (NICU), [C_reconstituted_] = 50 mg/mL. Then, ganciclovir must be diluted with a 5% glucose solution by the nurse before administration occurs at a concentration that does not exceed 10 mg/mL [23].

The prescribed dose of ganciclovir was 9 mg at the concentration of [C_prescribed_] = 5 mg/mL. To do this, the nurse takes 1 mL of [C_reconstituted_] and adds 9 mL of 5% dextrose in a 10 mL syringe. Then, she takes 3.6 mL of this solution and fills it up to 6 mL in a 10 mL syringe, which she administers to the patient at a rate of 3 mL/h [C_infused1_] to take account of the dead volume of the device’s tubing. The reconstitution protocol is detailed in the Supplementary Material Data.

**Figure 1:**
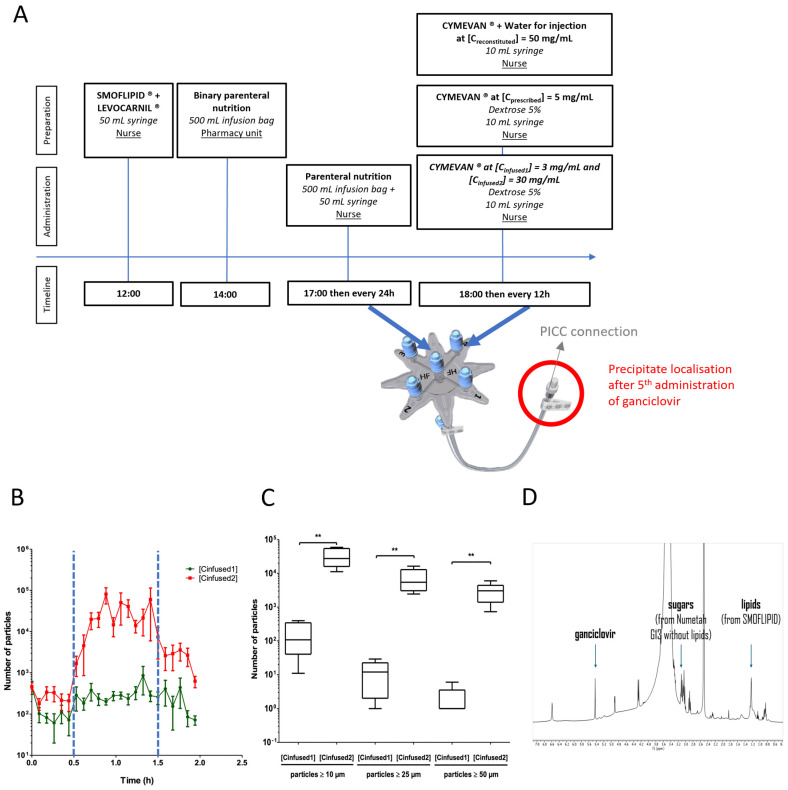
(**A**) Timeline of the preparation and administration of parenteral infusion drugs. (**B**) The particulate load observed during the infusion of PN and ganciclovir at [C_infused1_] (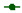) or ganciclovir at [C_infused2_] (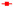). The blue dotted lines (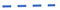) correspond to the start and the end of the ganciclovir infusion (t = 30 min and t = 1.5 h, respectively). The results are expressed as the mean ± Standard Deviation (SD) (*n* = 5). (**C**) The impact of the ganciclovir concentration on the particulate load. Comparisons of the particulate load were ≥10 µm, the particulate load was ≥25 µm, and the particulate load was ≥50 µm in PN + ganciclovir at [C_infused1_] = 3 mg/mL or PN + ganciclovir at [C_infused2_] = 30 mg/mL. The results are expressed as the median (range) (** *p* < 0.01 in a Mann–Whitney test, *n* = 5). (**D**) ^1^H nuclear magnetic resonance (NMR) spectra of the precipitate in deuterated DMSO (400 MHz, Bruker, Wissembourg, France) showing the presence of ganciclovir, and sugar and lipids from NUMETAH^®^ G13% without lipids and SMOFLIPID^®^, respectively, from identified representative peaks.

The ganciclovir infusion was administered as a one-hour intravenous infusion every 12 h, twice a day, while the parenteral nutrition (PN) was administered continuously and changed every 24 h (Figure 1A). This preparatory stage was carried out for each administration of ganciclovir. The obstruction of the PICC occurred during the fifth administration of ganciclovir.

Based on these data, an analysis of the literature identified the molecules potentially involved in the onset of PCIs. Two drugs were selected: ganciclovir and PN [24,25]. In fact, there is a theoretical PCI between ganciclovir and PN, but it normally occurs at higher concentrations (10 mg/mL) than those prescribed [24]. The type of PCI has not been described in the literature and remains unknown to this day.

A real-time reproduction of the administrations was carried out during 72 h in the laboratory. The exact same medical devices (Administration Device, PICC tubing, and syringe pumps) were used to reproduce concomitant perfusions. Concerning medicines, the administered drugs were ganciclovir and PN (Table 1) at the same concentrations and dosages described above. Replications of the administration schedule, therefore, included the continuous administration of PN and five administrations of ganciclovir, 12 h apart. After four administrations of ganciclovir to [C_infused1_], no PCI was detected. On the other hand, an experimental study was carried out without diluting the stock solution of ganciclovir after C_reconstituted_ ([C_reconstituted_] = 50 mg/mL). In this case, 3.6 mL was taken directly from the 10 mL syringe at C_reconstituted_ and not from the 10 mL syringe at the prescribed C_infused1_ (corresponding of a dilution to one-tenth of C_reconstituted_). The syringe was then topped up to 6 mL, thus leading to a C_infused2_ concentration. This situation mimicked forgetting to reconstitute to the 10^th^ between C_reconstituted_ and C_infused_. The obstruction was then immediately reproduced, with a macroscopic PCI on the PICC and at the distal end of the EDELVAISS^®^ Multiline Neo device, leading to discontinuation of the infusion and total obstruction of the catheter. Taking into account the infusion rate on the syringe pump, it has been shown that obstruction occurs as soon as the PN and ganciclovir meet.

A nuclear magnetic resonance (NMR) analysis was carried out to identify the composition of the obtained precipitate (analysis carried out in deuterated dimethylsulfoxide (DMSO) as a solvent, in which all the precipitate was soluble, with a 400 MHz Bruker). ^1^H NMR analysis of the nature of the precipitate revealed that it was composed of PN (especially sugars) and ganciclovir, confirming the hypothesis put forward in the literature review (Figure 1D). NMR spectra of the various elements are available in Supplementary Material Data.

Finally, a dynamic particle counter (QICPIC^®^, Sympatec GmbH, Clausthal-Zellerfeld, Germany) associated with a LIXELL^®^ (Sympatech GmbH, Clausthal-Zellerfeld, Germany) module estimated the number of particles (≥10 µm, ≥25 µm and ≥50 µm) infused into the patient during the infusion. A measurement was taken every 5 min during the 2 h infusion. PN and LEVOCARNIL^®^ infusion was 2 h long while the ganciclovir infusion was 1 h long, between t = 0.5 h and t = 1.5 h. During this PN + ganciclovir co-perfusion period, a high particulate load was present during PN + ganciclovir co-perfusion at [C_reconstituted_] compared with PN + ganciclovir co-perfusion at [C_prescribed_] (Figure 1B). The number of particles ≥10 µm, ≥25 µm, and ≥50 µm were significantly higher for the co-perfusion PN + ganciclovir at [C_reconstituted_] compared with the co-perfusion PN + ganciclovir at [C_prescribed_] (respectively, 175 ± 162 vs. 33,792 ± 20,117; *p* < 0.01; 12 ± 11 vs. 7498 ± 5622; *p* < 0.01; 1 ± 3 vs. 3532 ± 1657; *p* < 0.01, Mann–Whitney, *n* = 5 (Figure 1C).

The hypothesis of the occurrence of the PCI retained by our analysis is that of an incorrect dilution of ganciclovir, leading to a concentration at which the PCI appears at [C_infused2_] = 30 mg/mL. A first reconstitution was made from the powder vial to the stock solution, but the following one-tenth dilution was probably omitted. Unfortunately, medication errors are a non-negligible phenomenon in neonatal critical care, particularly due to the lack of special formulations adapted the specific needs of preterm neonates with low doses [26].

## 3. Discussion

PCIs remain a major risk associated with healthcare, even if further studies are needed to find out more about the level of exposure to PCIs and their clinical implications. It can induce organ failure, in particular, pulmonary toxicity and systemic inflammatory response syndrome [27]. Depending on the size and the form of the particles, they can also induce renal embolism [9]. Therefore, it is important to identify them and limit them as much as possible.

The strategies outlined and assessed are filtration, use of multi-lumen devices, purging of infusion lines, incompatibility tables and databases, and the use of standard operating procedures [14,28]. Here, the multi-lumen device did not prevent this PCI or its consequences. The precipitate appeared in the fifth dose because the first four doses had been correctly prepared: the double dilution had been carried out. In fact, there was a common volume at the tip, and it was also connected to terminal administration devices (PICCs), which further disrupted the flow. The use of an EDELVAISS^®^ Multiline Neo device should not lead to a reduction in vigilance on the part of users: it remains essential to identify potential PCIs and to adapt the set-up and administration methods. In vitro compatibility tests and training programmes on PCIs remain essential to increasing the security of parenteral drug administration and the knowledge of how to anticipate the occurrence of these PCIs [5].

Here, the child had to undergo a new invasive procedure involving the insertion of a new PICC line, but fortunately, there were no other clinical consequences attributed to this adverse event associated with care. The analysis of the prescription must be carried out systematically, including PN, to assess the presence of any PCI in order to adapt the level of prevention and vigilance of the entire care team and to reduce the risk of adverse events associated with co-administered drug infusions. Software can be useful to help caregivers due to the large number of prescribed drug lines [29]. This also means more research on PCI, as the data are not always available. PCI detection software will be introduced in pilot high-risk departments in our establishment. It will help to prevent PCIs and can also be used in the event of a PCI to analyse the molecules potentially involved in the prescriptions.

This study has various limitations. The first concerns particle counting. It could not be carried out in the presence of SMOFLIPID^®^ because this emulsion opacifies the mixture and makes it impossible to count non-visible particles. Therefore, it is possible that PCIs may occur between ganciclovir and SMOFLIPID^®^, but this was not possible to demonstrate by using this technique.

In addition, there is a PCI between ganciclovir and two LEVOCARNIL^®^ excipients: sodium methyl parahydroxybenzoate and sodium propyl parahydroxybenzoate. Both are molecules in the paraben family. The recommendations for the preparation of ganciclovir advise against reconstituting ganciclovir with water containing parabens, as there is a risk of producing a precipitate. In the case of this study, the very small quantity of LEVOCARNIL^®^ prepared in SMOFLIPID^®^ (0.1 mL, sufficient quantity to 30 mL) did not allow any visible and/or non-visible PCI to occur.

As mentioned above, the exact mechanism by which a PCI occurs is not known. The typology of this PCI and the mechanisms behind the precipitate formation are being investigated as part of another study currently underway. As far as NMR is concerned, our work does not allow us to characterise the actual composition of the precipitate, which will always remain uncertain, but the use of this exploratory method has enabled us to identify the elements involved.

It was not possible to determine precisely how many minutes it took for the PCI to be detected and, therefore, the precise quantity that the child received (between a few milligrams and a maximum of 90 mg).

Finally, this study is a retrospective approach following the occurrence of an adverse event associated with care. All the events were reconstructed on the basis of information available in the prescription software and exchanged with the medical team. Therefore, there is a memory bias inherent in this type of study.

Regardless of age, ganciclovir is essential in the management of CMV infection disease, particularly in the transplantation setting [30]. Practitioners should be aware of the potential PCIs that may occur with use. Similarly, practitioners should consider PN as a drug that needs to be included in the search for PCIs. Its composition, high amino acid levels, and pH, in this case 5.5, make it a source of numerous incompatibilities.

As it only occurred at a high concentration (due to an incorrect dilution factor) reported in the literature [24,25], it could simply be due to insolubility, for example, due to salting. Our results are consistent with the literature data. Indeed, no PCIs are observed at ganciclovir concentrations ranging from 0.83 to 5 mg/mL, and PCI is observed beyond 10 mg/mL [31]. However, further studies are needed, in particular, to define the concentration threshold at which PCIs involving ganciclovir begin to appear. The search for PCI between ganciclovir and other molecules frequently associated with clinical practice also needs to be developed, given its high frequency of use.

## 4. Conclusions

Multi-lumen infusion devices help reduce but do not entirely eliminate, the risk of PCIs, especially in cases of preparation errors. Despite their use, strict vigilance remains essential, particularly in prescription analysis and reconstitution procedures. Communication with carers regarding multi-lumen infusion devices must fully integrate the notion of “reducing” the risk of PCIs and not “eliminating” it. Studies are underway on PCIs between ganciclovir and parenteral nutrition to better characterise the mechanisms behind the formation of the precipitate, the nature of the precipitate, and the critical concentration thresholds.

## Figures and Tables

**Table 1:** Drug specialities administered to the preterm infant.

	Pharmaceutical Form	Drug Specialities (Concentration) Active Substances	Flow Rate	Volume or Dose	Prescription
**Parenteral nutrition (PN)**	Parenteral IV solution Binary parental nutrition 500 mL Infusion Bag	NUMETAH^®^ G13% Binary PN	6.4 mL/h	139 mL	1/day
		Water for Injection		37 mL	
		CALCIUM GLUCONATE^®^ 10% (9.1 mg/mL) Calcium gluconate		70 mg	
		PHOCYTAN^®^ (10.23 mg/mL) Disodium glucose-1-phosphate tetrahydrate		70 mg	
		CERNEVIT^®^ Vitamin complex		1.5 mL	
		ZINC INJECTABLE^®^ (1 mg/mL) Zinc gluconate		0.4 mL	
		JUNIMIN^®^ Oligo-elements		1.5 mL	
	Parenteral IV emulsion 50 mL syringe	SMOFLIPID^®^ (200 mg/mL) Lipid emulsion	1.25 mL/h	30.5 mL	1/day
		LEVOCARNIL^®^ (200 mg/mL) Levocarnitine		15 mg	
**Anti-infectious**	Parenteral IV solution 50 mL syringe	CYMEVAN^®^ 500 mg Ganciclovir	3 mL/h	9 mg	2/day

Drug specialties manufacturer information: NUMETAH^®^ G13% Binary PN (Baxter S.A.S., Guyancourt, France); CALCIUM GLUCONATE^®^ 10% (Laboratoire Aguettant, Lyon, France); PHOCYTAN^®^ (Laboratoire Aguettant, Lyon, France); CERNEVIT^®^ (Baxter S.A.S., Guyancourt, France); ZINC INJECTABLE^®^ (Laboratoire Aguettant, Lyon, France); JUNIMIN^®^ (Laboratoire Aguettant, Lyon, France); SMOFLIPID^®^ (Fresenius Kabi France, Sevres, France); LEVOCARNIL^®^ (Alfasigma France, Issy-les-Moulineaux, France); CYMEVAN^®^ (Cheplapharm Arzneimittel GmbH, Greifswald, Germany).

## Data Availability

Data are contained within the article and the Supplementary Materials.

## Supplementary Materials

The following supporting information can be downloaded at: https://www.mdpi.com/article/10.3390/ph18050626/s1, Figure S1: NMR spectra of the various components, Figure S2: Preparation protocol for ganciclovir

## Abbreviations

CMV: Cytomegalovirus, PCI: Physico-Chemical Incompatibility, NMR: Nuclear Magnetic Resonance, PICC: Peripheral Inserted Central Catheter, PN: Parenteral Nutrition.
