# Supplementary figures and images for: Drug Incompatibilities and Complex Assemblies: Let Us Remain Vigilant!
Source: Pharmaceuticals (Basel). 2025 Apr 25;18(5):626. doi: 10.3390/ph18050626 (PMC12114821; doi:10.3390/ph18050626)

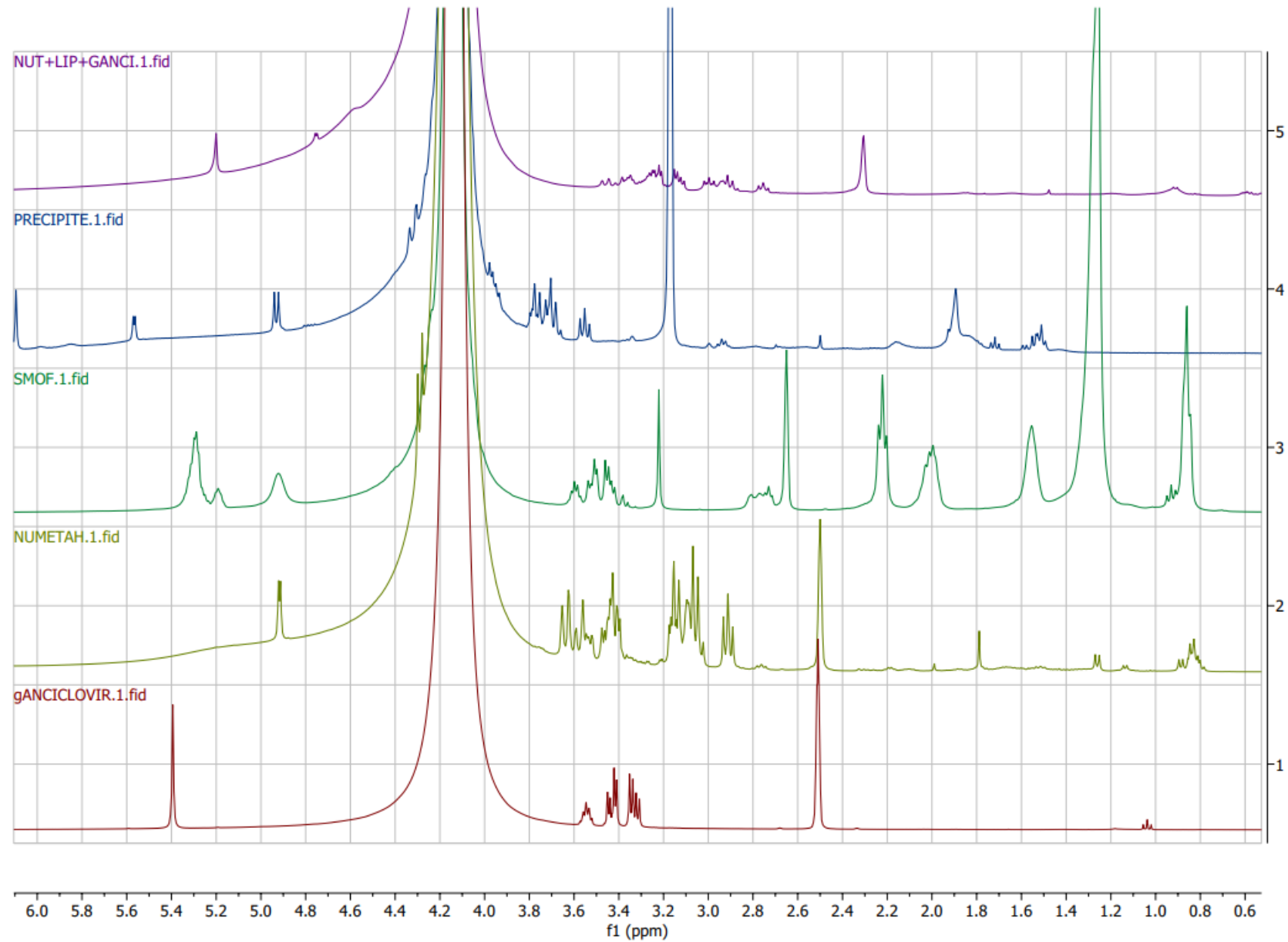

Figure S1 : NMR spectra of the various components

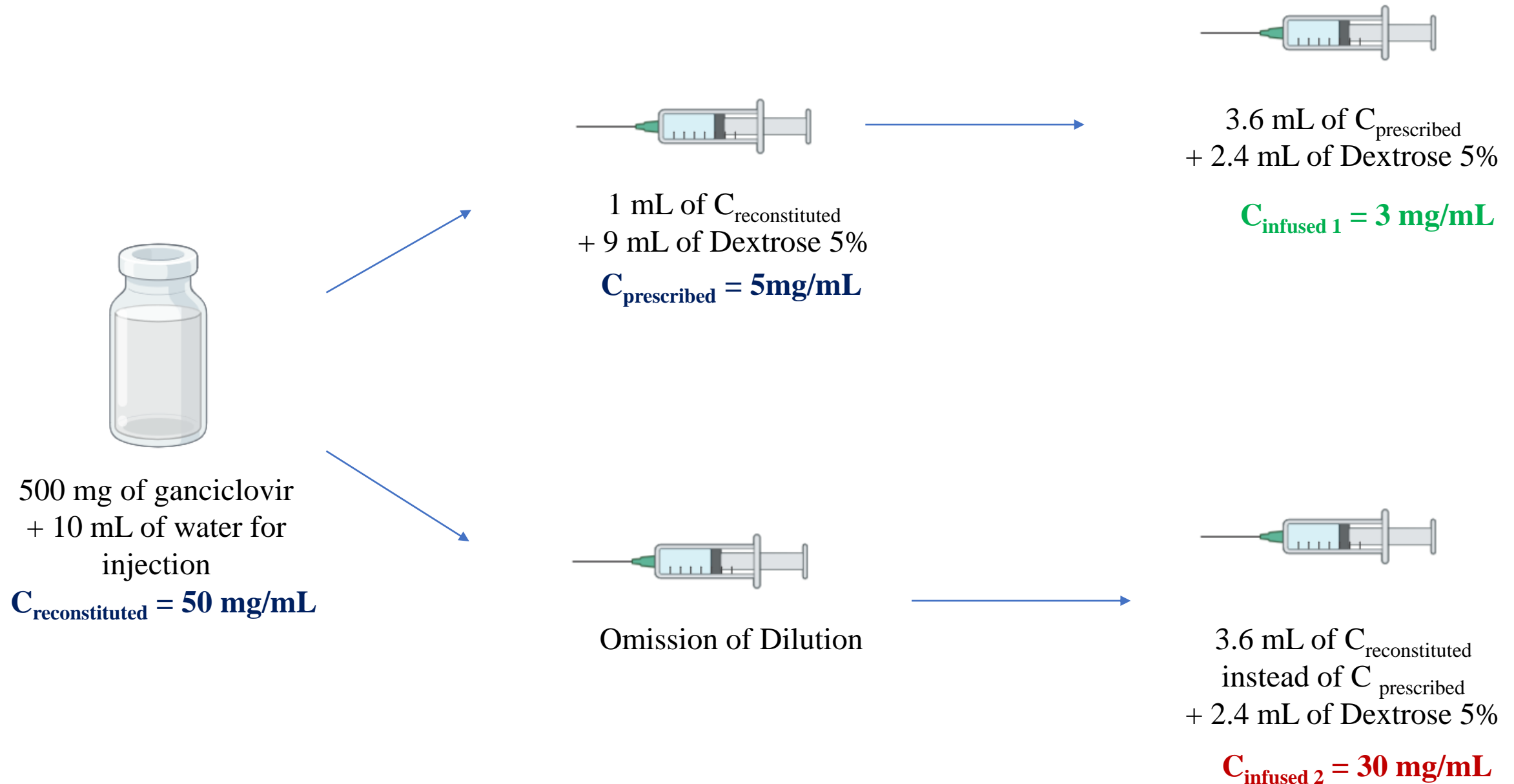

Figure S2 : Preparation protocol for ganciclovir

Supplement: Supplementary file 1 [file pharmaceuticals-18-00626-s001.zip › pharmaceuticals-3468456-supplementary.pdf]
